# DNA Methylation Profiling Enables Accurate Classification of Nonductal Primary Pancreatic Neoplasms

**DOI:** 10.1016/j.cgh.2024.02.007

**Published:** 2024-02-20

**Authors:** Anna Vera D. Verschuur, Wenzel M. Hackeng, Florine Westerbeke, Jamal K. Benhamida, Olca Basturk, Pier Selenica, G. Mihaela Raicu, I. Quintus Molenaar, Hjalmar C. van Santvoort, Lois A. Daamen, David S. Klimstra, Shinichi Yachida, Claudio Luchini, Aatur D. Singhi, Christoph Geisenberger, Lodewijk A. A. Brosens

**Affiliations:** 1 Department of Pathology, University Medical Center Utrecht, Utrecht University, Utrecht, The Netherlands; 2 Department of Pathology and Laboratory Medicine, Memorial Sloan Kettering Cancer Center, New York, New York; 3 Department of Pathology, St Antonius Hospital and Pathology DNA, Nieuwegein, The Netherlands; 4 Department of Surgery, Regional Academic Cancer Center Utrecht, University Medical Center Utrecht Cancer Center and St. Antonius Hospital, Nieuwegein, The Netherlands; 5 Paige.AI, New York, New York; 6 Department of Cancer Genome Informatics, Graduate School of Medicine, Osaka University, Osaka, Japan; 7 Department of Diagnostics and Public Health, Section of Pathology, University of Verona, Verona, Italy; 8 Department of Pathology, University of Pittsburgh School of Medicine, Pittsburgh, Pennsylvania; 9 Institute of Pathology, LMU University, Munich, Germany; 10 Department of Pathology, Radboud University Medical Center, Nijmegen, The Netherlands

**Keywords:** Pancreatic Neuroendocrine Tumor, Acinar Cell Carcinoma, Solid Pseudopapillary Neoplasms, Pancreatoblastoma, Pancreatic Ductal Adenocarcinoma, DNA Methylation, Tumor Classification

## Abstract

**BACKGROUND & AIMS:**

Cytologic and histopathologic diagnosis of non-ductal pancreatic neoplasms can be challenging in daily clinical practice, whereas it is crucial for therapy and prognosis. The cancer methylome is successfully used as a diagnostic tool in other cancer entities. Here, we investigate if methylation profiling can improve the diagnostic work-up of pancreatic neoplasms.

**METHODS:**

DNA methylation data were obtained for 301 primary tumors spanning 6 primary pancreatic neoplasms and 20 normal pancreas controls. Neural Network, Random Forest, and extreme gradient boosting machine learning models were trained to distinguish between tumor types. Methylation data of 29 nonpancreatic neoplasms (n = 3708) were used to develop an algorithm capable of detecting neoplasms of non-pancreatic origin.

**RESULTS:**

After benchmarking 3 state-of-the-art machine learning models, the random forest model emerged as the best classifier with 96.9% accuracy. All classifications received a probability score reflecting the confidence of the prediction. Increasing the score threshold improved the random forest classifier performance up to 100% with 87% of samples with scores surpassing the cutoff. Using a logistic regression model, detection of nonpancreatic neoplasms achieved an area under the curve of >0.99. Analysis of biopsy specimens showed concordant classification with their paired resection sample.

**CONCLUSIONS:**

Pancreatic neoplasms can be classified with high accuracy based on DNA methylation signatures. Additionally, non-pancreatic neoplasms are identified with near perfect precision. In summary, methylation profiling can serve as a valuable adjunct in the diagnosis of pancreatic neoplasms with minimal risk for misdiagnosis, even in the pre-operative setting.

## Graphical Abstract

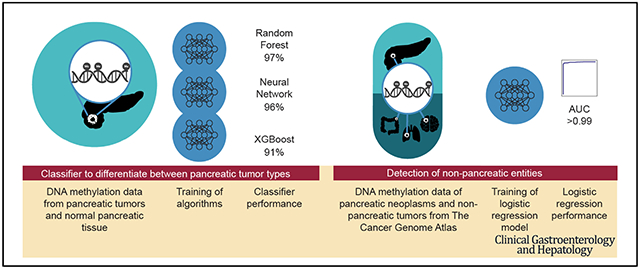

Around 90% of pancreatic neoplasms are ductal adenocarcinomas (PDACs); the remainder is of non-ductal origin, including pancreatic neuroendocrine neoplasms (pancreatic neuroendocrine tumor [PNET] and pancreatic neuroendocrine carcinoma [PNEC]), acinar cell carcinoma (ACC), solid pseudopapillary neoplasms (SPN), and pancreatoblastoma (PB), accounting for 5%, 2%, 1%, and <1% of cases, respectively.^1^ These tumors differ significantly in biology and clinical outcomes. For instance, SPNs rarely metastasize and have a 95% 5-year survival rate,^2^ whereas PNETs, ACCs, and PBs show 5-year survival rates of 38%–59%.^3–5^ Additionally, these tumors require different treatment. ACCs are typically treated by a combination of surgical resection, chemotherapy, and radiotherapy^6^; SPNs are cured by surgical resection^7^; and PNET management varies from resection to active surveillance depending on such factors as hormone production, tumor size, grade, and stage.^8,9^ Hence, accurate diagnosis is crucial for therapeutic decision making, but ACCs, SPNs, and PNETs can present diagnostic challenges because of overlapping cytologic, histologic, and immunohistochemical features, particularly when dealing with limited tissue samples, such as preoperative fine-needle aspirates (FNAs) or fine-needle biopsies (FNBs).^1,10–12^

To improve diagnosis, there is a growing trend in using whole genome methylation-based diagnostic models for tumor classification,^13–17^ with the World Health Organization recommending its routine application in central nervous system tumors.^18^ DNA methylation, a covalent modification of cytosine residues regulating gene expression, is known for its high celltype specificity, making it effective in differentiating between tumor types.^17,19,20^ Previous studies identified the origin of different gastrointestinal (neuroendocrine) tumors based on methylation profiles, including those originating in the pancreas, indicating potential utility in non-ductal pancreatic neoplasms.^21–23^

This study aimed to facilitate and improve preoperative and postoperative diagnosis of non-ductal pancreatic neoplasms, by developing a machine learning classifier on a well-curated collection of pancreatic neoplasms. Additionally, we aimed to make the classifier capable of detecting non-pancreatic entities.

## Materials and Methods

### Study Overview

**Figure 1.**
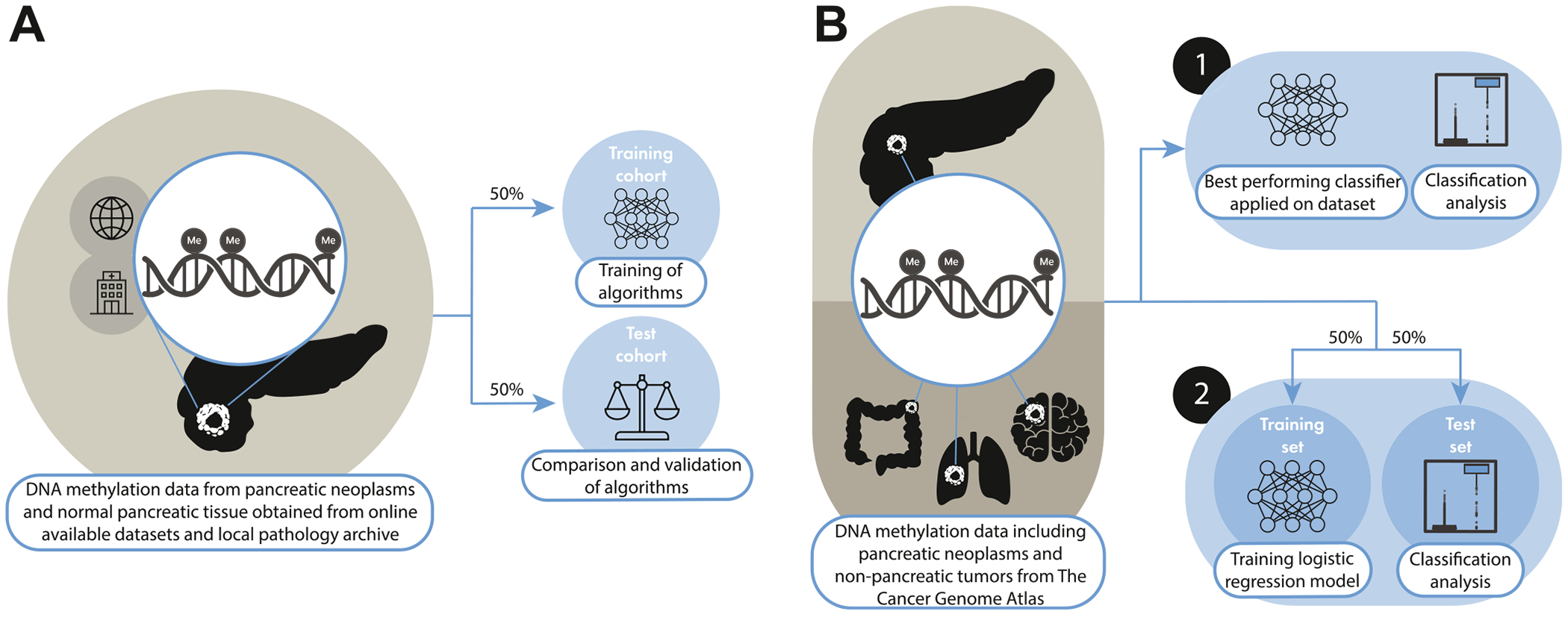
Study methods for classifier development and detection of non-pancreatic entities. A cohort of pancreatic neoplasms and pancreatic controls, comprising publicly available and in-house DNA methylation data, was randomly divided into training and test cohort stratified by tumor type (50:50 ratio). Neural Network, Random Forest, and extreme gradient boosting models were trained on the training cohort and evaluated in the test cohort (*A*). A cohort of pancreatic and non-pancreatic neoplasms was used to (1) evaluate the Random Forest’s performance in detecting non-pancreatic tumors and to (2) optimize nonpancreatic tumor detection using logistic regression (*B*).

Methylation data from 20 normal pancreatic tissues and 301 major primary pancreatic tumor types were collected to develop a classification algorithm (Table 1).^22–28^ Samples were randomly split into training and test cohorts stratified by tumor type (50:50 ratio; Figure 3A). Neural Network (NN), Random Forest (RF), and extreme gradient boosting (XGB) models were trained on the training cohort and evaluated in the test cohort (Figure 1A). Considering that the models are specifically trained to classify only the tumor types present in their training data, we enhanced the model with the ability to identify neoplasms of non-pancreatic origin (eg, tumors types it is not trained on). To do so, a logistic regression model using the pancreatic dataset (n = 321) supplemented with non-pancreatic neoplasms (n = 3708) was trained based on the RF output (Figure 1B). Furthermore, we performed 3 additional analyses with (1) mixed neuroendocrine-non-neuroendocrine neoplasm (MiNEN, n = 7), (2) matched primary and metastatic cases (n = 5), and (3) matched FNA and resection specimens (n = 4). Details on patients and samples, DNA extraction and whole genome DNA methylation analysis, preprocessing, quality control and batch effect, unsupervised analysis, classifier development and validation, and additional analysis are described in Supplementary Methods, Supplementary Table 1, and Supplementary Figure 1.

### Code Availability

All analyses were performed in RStudio version 2023.03.1 based on the statistical language R version 4.3.0, using the tidyverse software collection for data wrangling and ggplot for visualization. Code used for the generation of results and figures can be accessed at https://github.com/averschuur/pancreas.

## Results

### Pancreatic Dataset

**Figure 2.**
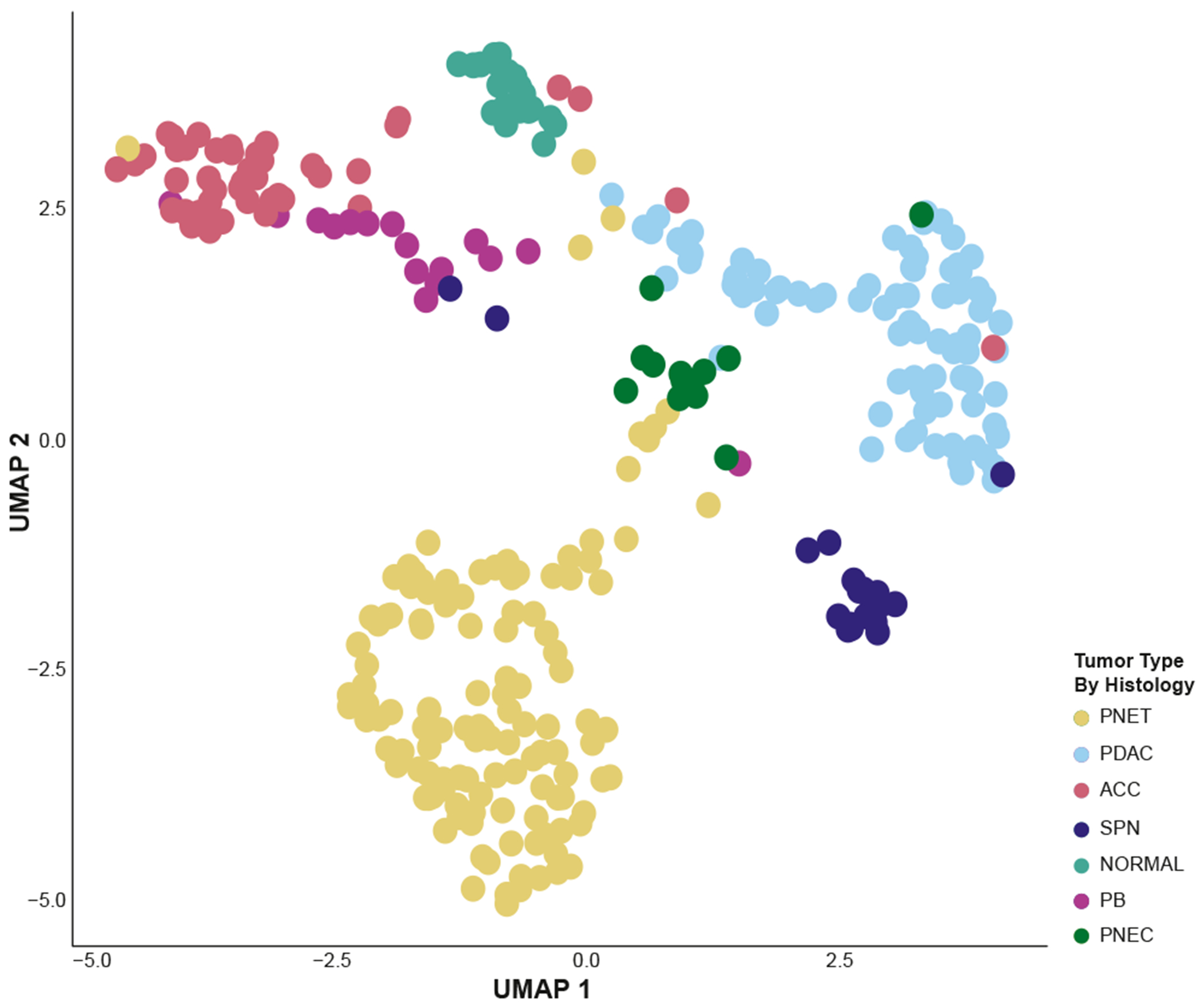
Visualization of methylation profiles of the pancreatic dataset using UMAP dimensionality reduction.

The entire study cohort comprises 301 primary pancreatic tumors including 124 PNETs, 13 PNECs, 46 ACCs, 20 SPNs, 16 PBs, 82 PDACs, and 20 normal pancreas controls (Table 1). Dimensionality reduction with uniform manifold approximation and projection (UMAP) showed clustering predominantly by tumor type (Figure 2). Analysis of average methylation distribution, bisulfite conversion efficiency, and tumor purities based on ESTIMATE and ABSOLUTE scores across tumor types revealed lower purity in PDACs and lower genome-wide methylation in PBs (Supplementary Figure 2A–H). Projecting different sample preservation types onto the UMAP demonstrated no difference in clustering between fresh-frozen and formalin-fixed, paraffin embedded tissue (Supplementary Figure 3). Batch effect analyses revealed small but consistent differences regarding beta values distribution and conversion scores across tumor types (Supplementary Figure 4A–T).

### Classifier Development and Performance

**Figure 3.**
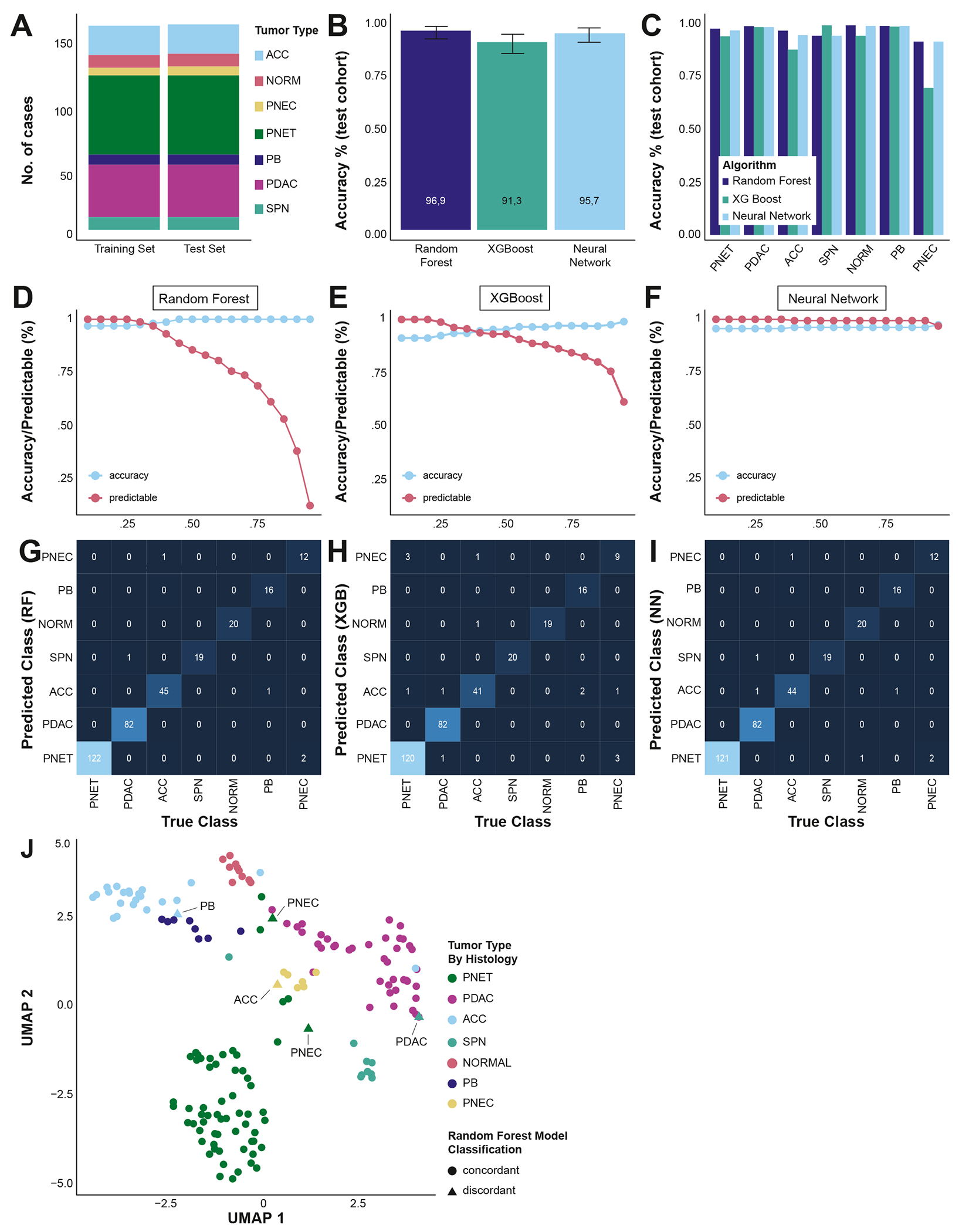
Development, comparison, and validation of DNA methylation–based classifier. Sample counts for each tumor type in the training and test sets (*A*). The classifiers achieved accuracies of 96.9% (Random Forest), 91.3% (XGB), and 95.7% (Neural Network) in the test cohort (*B*). Evaluation of classifier accuracies by tumor type shows that the Random Forest classifier consistently outperforms the Neural Network and XGB algorithms (*C*). Threshold analysis of Random Forest (*D*), XGB (*E*), and Neural Network (*F*) probability scores demonstrates that the Random Forest model achieves the highest accuracy in most cases. Confusion matrices for Random Forest (*G*), XGB (*H*), and Neural Network (*I*) showing discordant classifications per tumor type. *UMAP* visualization of the random forest classification results within the test cohort demonstrates that 2 out of 5 cases actually cluster with the tumor type assigned by the classifier (*J*).

When assessing the classifiers without any cutoffs and assigning each sample to the category with the highest probability score, classifiers achieved accuracies of 96.9% (RF), 91.3% (XGB), and 95.7% (NN) in the test cohort (Figure 3B). The RF model consistently outperformed other models across all tumor types, except for SPN (Figure 3C). The accuracies of the models were not significantly different (*P* = .40; 1-way analysis of variance).

Applying a threshold with minimum classification scores of 0.45 (RF), 0.95 (XGB), and 0.80 (NN), improved accuracies to 100%, 99%, and 97%, respectively (Figure 3D–F). With the same threshold, classification was possible for 87% (RF), 62% (XGB), and 99% (NN) of samples. Adjusting the RF score cutoff to 0.30 resulted in 97.5% accuracy while retaining 98.8% of samples, comparable with the maximum accuracy of NN (97.4%) while retaining a slightly lower proportion of samples (96.9%). Taken together, the RF model without any cutoffs demonstrated the highest accuracy in the highest proportion of samples.

Furthermore, the confusion matrices of the RF, XGB, and NN classifiers give an overview of misclassified cases per tumor type (Figure 3G–I). Projecting RF classification results within the test cohort onto UMAP demonstrates that 2 out of 5 cases cluster with the tumor type assigned by the classifier (Figure 3J). In summary, RF achieved almost 97% accuracy in an independent test cohort with similar results across different tumor types, with potential for further improvement using score cutoffs.

### Detection of Non-pancreatic Neoplasms

**Figure 4.**
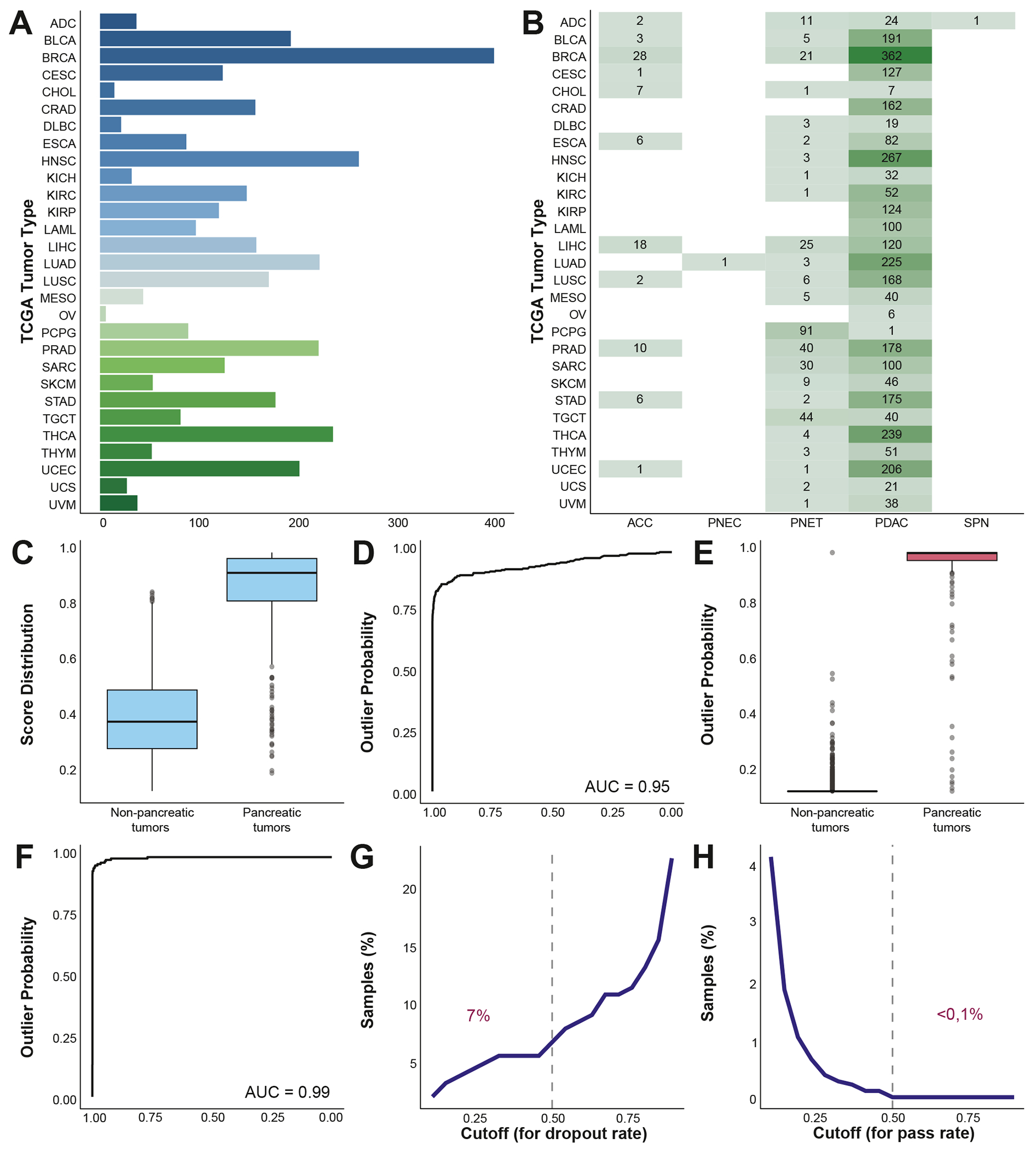
Detection of non-pancreatic neoplasms. Sample counts per tumor type in the non-pancreatic dataset (*A*). The Random Forest model classified all non-pancreatic tumors as PDAC, PNET, ACC, PNEC, or SPN (*B*). Random Forest score distribution of non-pancreatic and pancreatic neoplasms (*C*). Receiver operating characteristic (ROC) curves based on Random Forest distribution scores show an outlier probability with area under the curve (AUC) of 0.95 (*D*). A second algorithm (logistic regression) was developed to identify non-pancreatic neoplasms, and its probability scores were lower for non-pancreatic neoplasms (*E*). ROC curve based on logistic regression shows an improved AUC of >0.99 (*F*). Threshold analysis shows real pancreatic samples considered as non-pancreatic neoplasms (dropouts) (*H*) and non-pancreatic neoplasms classified as pancreatic neoplasms (pass rate) (*I*). At a cutoff of 0.5, dropout is 7% and pass rate <0.1%. The Cancer Genome Atlas (TCGA) abbreviations: ADC, adrenocortical carcinoma; BLCA, bladder urothelial carcinoma; BRCA, breast invasive carcinoma; CESC, cervical squamous cell carcinoma and endocervical adenocarcinoma; CHOL, cholangiocarcinoma; CRAD, colorectal adenocarcinoma; DLBC, lymphoid neoplasm diffuse large B-cell lymphoma; ESCA, esophageal carcinoma; HNSC, head and neck squamous cell carcinoma; KICH, kidney chromophobe; KIRC, kidney renal clear cell carcinoma; KIRP, kidney renal papillary cell carcinoma; LAML, acute myeloid leukemia; LIHC, liver hepatocellular carcinoma; LUAD, lung adenocarcinoma; LUSC, lung squamous cell carcinoma; MESO, mesothelioma; OV, ovarian serous cystadenocarcinoma; PCPG, pheochromocytoma and paraganglioma; PRAD, prostate adenocarcinoma; SARC, sarcoma; SKCM, skin cutaneous melanoma; STAD, stomach adenocarcinoma; TGCT, testicular germ cell tumors; THCA, thyroid carcinoma; THYM, thymoma; UCEC, uterine corpus endometrial carcinoma; UCS, uterine carcinosarcoma; UVM, uveal melanoma.

Considering that the models are specifically trained to classify pancreatic neoplasms, for diagnosis it is crucial to detect neoplasms of non-pancreatic origin. First, we applied the RF model in an extended cohort of 3708 samples encompassing 29 nonpancreatic neoplasms including those known to metastasize to the pancreas (Figure 4A). Out of 7 possible categories, the RF model classified all non-pancreatic tumors as either PDAC, PNET, ACC, SPN, or PNEC (Figure 4B). Non-pancreatic neoplasms received significantly lower probability scores compared with pancreatic neoplasms and the receiver operating characteristic analysis of RF probability scores revealed an area under the curve of >0.95 (Figure 4C and D). Logistic regression using RF scores and classification results as input was used to identify non-pancreatic neoplasms. The resulting probability scores were lower for non-pancreatic neoplasms (Figure 4E) and receiver operating characteristic analysis showed an improved area under the curve of >0.99 compared with RF scores alone (Figure 4F). At a cutoff of 0.5, 7% of pancreatic tumors were classified as non-pancreatic, whereas <0.1% non-pancreatic neoplasms were classified as originating from the pancreas (Figure 4G and H). This additional safety measure can be used to flag samples of non-pancreatic origin. Excluding flagged samples from the test cohort improved classifier performance to 97.9% (RF), 93.6% (XGB), and 97.9% (NN).

### Classifier Application in Histopathologic Challenging and Misdiagnosed Clinical Cases

**Figure 5.**
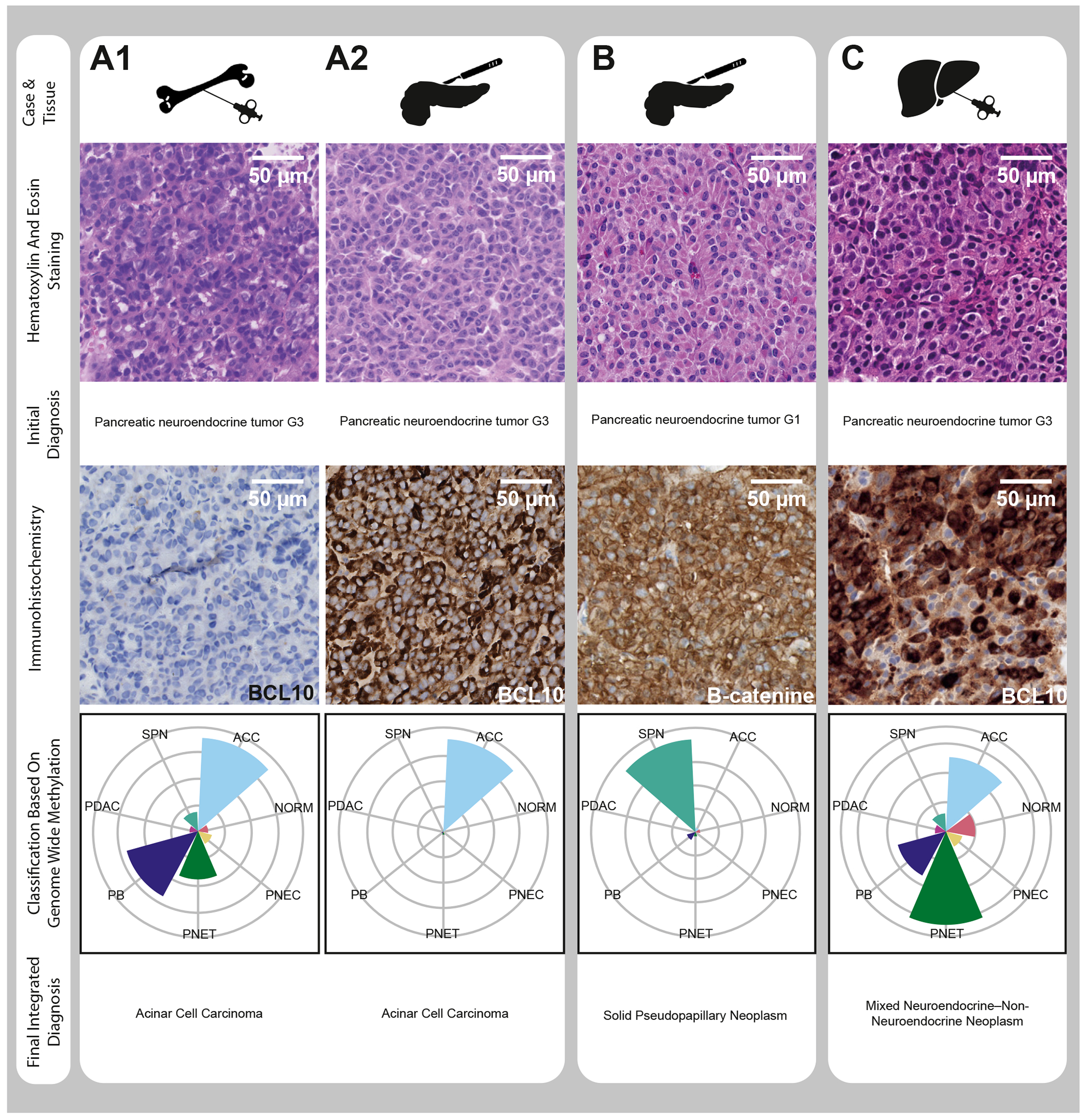
Classifier application in histopathologically misdiagnosed and challenging clinical cases. Case A includes a spine biopsy (case A1) and resection specimen of the primary pancreatic lesion (case A2) of a patient with moderate radioactive depositions in the pancreas and spine on ^68^Ga-*DOTATOC* PET/CT suggesting metastasized PNET. Based on positive neuroendocrine markers and negative ACC marker BCL10, the case was signed out as metastatic PNET grade 3. Histopathologic analysis of the subsequent pancreatic resection specimen showed positivity of BCL10 changing the diagnosis to ACC. The Random Forest model classified both the bone biopsy and the pancreatic lesion as ACC. Case B, initially misdiagnosed as a PNET, was later reevaluated as a SPN on revision at a tertiary referral center. This reclassification was based on additional immunohistochemistry studies, which showed positivity for SPN markers (β-catenin, vimentin, and CD10). The Random Forest model confirmed the case as SPN. Case C is a liver biopsy classified as MiNEN (mixed ACC-PNET) based on expression of both neuroendocrine markers and ACC marker BCL10 in approximately half of the tumor cells. The Random Forest classification was divergent and showed a high probability score for ACC and PNET, consistent with the histopathologic suspicion of MiNEN.

To investigate the added value of methylation profiling, we retrospectively applied the RF classifier on 3 cases that were misdiagnosed (cases A1 and B) or difficult to classify (case C) during initial histopathologic assessment (Figure 5).

The first case, a ^68^Ga-DOTATOC PET/CT, revealed a moderately radioactive deposition in the pancreas and spine, suggesting metastasized PNET. Histopathologically, the differential diagnoses of the spinal cord biopsy were PNET and ACC. Because neuroendocrine markers were positive and ACC marker BCL10 was negative (Figure 5, case A1), the case was signed out as metastasis of a PNET grade 3 and the patient underwent resection of the primary tumor in the pancreas. Histopathologic analysis of the resection specimen showed comparable immunohistochemistry with the biopsy but, surprisingly, strong BCL10 expression, changing the diagnosis to ACC (Figure 5, case A2). Methylation profiling by the RF model classified both the bone biopsy and the pancreatic lesion as ACC.

The second case was initially classified as PNET but histopathologic revision at a tertiary expert center raised doubt about this diagnosis. Based on additional immunohistochemistry of SPN markers (*β*-catenin, vimentin, and CD10), the case was reclassified as SPN (Figure 5, case B). The RF model also classified the case as SPN.

The third case was a liver biopsy that showed a tumor with varying neuroendocrine marker expression and BCL10 positivity in about half of the tumor cells (Figure 5, case C). The differential diagnosis included a pancreatic neuroendocrine neoplasm (PNET G3 or PNEC), ACC with neuroendocrine differentiation, or mixed acinar-neuroendocrine neoplasm. Molecular immunohistochemistry showed, among other things, ATRX loss and TSO500 sequencing TruSight Oncology (Illumina) revealed mutations in *NRAS* (p.Q61R), *RAD50*, and *ATM*. Taken together, although noted that this is a difficult tumor to classify, the case was signed out as a MiNEN (PNET G3-ACC). The RF classification yielded divergent results, with high probability scores for both ACC and PNET, aligning with the suspicion of a MiNEN. Because of these divergent probability scores, we analyzed 7 additional primary MiNENs that obtained corresponding divergent classification results and higher entropy compared with pancreatic tumors (Supplementary Figure 5A–E).

### Additional Analysis on Paired Cases

First, 4 primary tumors with matched metastases were analyzed: 3 liver metastases from 2 primary PNETs, 1 liver metastasis from a MiNEN, and 1 bone metastases from an ACC (Figure 5, cases A1 and A2). High correlations were observed between the matched samples compared with other cases, and metastases and primary lesions were classified identically (Supplementary Figure 6A–C). Second, 4 paired preoperative FNA cytology and resection specimens were analyzed. The EPIC array successfully processed all FNA cases, and there were strong correlations between matched samples compared with other cases. Identical classifications were observed in all matched cases (Supplementary Figure 7A–C).

## Discussion

This proof-of-concept study effectively uses DNA methylation profiling to address the diagnostic challenge of distinguishing between different primary pancreatic tumors. After benchmarking the performance of 3 state-of-the-art machine learning models (NN, XGB, RF) on a comprehensive cohort spanning all relevant pancreatic tumor types, the RF model emerged as the best classifier with 96.9% accuracy. Because the models’ classification is limited to classify tumor types present in their training data, and to account for metastases to the pancreas, we developed a second algorithm based on the pancreatic dataset supplemented with non-pancreatic neoplasms. This approach reduced the probability of a false pancreatic neoplasm diagnosis to less than 1 in 1000 cases, whereas 7% of true pancreatic tumors were classified as non-pancreatic. Considering the rare occurrence of metastases to the pancreas and the artificial case mix, the actual risk of a false pancreatic tumor diagnosis is likely much lower.

The pancreatic-tumor classifier in this study is highly accurate, comparable with methylation-based classifiers for other tumor types (95.1% for brain tumors,^13^ 99.9% for sarcoma,^15^ 96% for squamous cell carcinoma,^14^ and 89% for head and neck squamous cell carcinoma^16^). Although RF models have traditionally been successful and widely used algorithms, recent studies suggest superiority of other models, such as NN, Support Vector Machines, and LOGREG.^13–16,21^ In this study, the performance of the 3 models was not significantly different. Nonetheless, adjusting cutoffs for minimum probability scores demonstrated that RF achieved higher accuracies for most of the cases. Its probability score distribution makes it especially well-suited for detecting nonpancreatic entities, making RF the preferred model in this study.

This study underscores the benefits of methylation profiling in diagnosing pancreatic tumors in several ways. First, the algorithm presented here effectively distinguishes between different pancreatic tumors, even when they exhibit overlapping signatures in unsupervised analysis, such as PB and ACC, which is likely caused by their similar epigenetic profiles linked to acinar differentiation.^22^ Our machine learning model correctly classified 30 out of 31 ACC and PB cases in the test cohort, indicating that dissimilarities between these tumors are significant enough to enable differentiation through DNA methylation. Of note, because of existing diagnostic challenges and the impact of sampling bias, methylation profiling alone is currently not considered suitable for classification of potential MiNEN cases.

Second, this classifier assists in excluding other tumor types in the differential diagnosis of pancreatic lesions. The non-pancreatic dataset includes primary neoplasms known to metastasize to or directly invade the pancreas, such as renal cell, lung, breast, colonic, and gastric cancer.^29,30^ This is valuable in excluding the possibility of metastasis to the pancreas. Additionally, the presence of cholangiocarcinoma in the non-pancreatic dataset helps exclude periampullary tumors from consideration. Although it would have been interesting to include more periampullary tumors, such methylation data were unavailable from public resources.

Lastly, DNA methylation profiling can aid diagnosis of FNA/FNB samples of primary tumors and metastases, particularly in challenging cases with insufficient material for additional immunohistochemistry, technical artefacts (ie, caused by decalcification, or cellular distortion). This study demonstrates that the RF model accurately classified all FNA/FNB samples. Thereby, it correctly classified the preoperative biopsy of a case affected by technical artifacts (case A), highlighting the potential robustness of DNA methylation profiling in various sample conditions, mitigating potential diagnostic errors.

Nonetheless, using publicly available DNA methylation data has limitations. First, our dataset had limited data on PB, SPN, and PNEC from only a few studies, possibly contributing to slightly lower model performance in these tumor types (Figure 3C). Second, we were unable to verify the histopathologic diagnoses from the publicly available DNA methylation data. Most samples that received a discordant classifier classification clustered with the corresponding tumor type. Although UMAP should not be considered a definitive means to classify samples, it suggests that at least in several cases, methylation profiling may have identified misdiagnosed samples. This has been noted previously for PDACs of The Cancer Genome Atlas cohort, which were later shown to actually represent PNETs.^31,32^ Because of a lack of additional data this hypothesis cannot be proven. Nonetheless, these dissimilarities highlight the potential value of methylation profiling as a second layer of information.

This study again demonstrates the potential of epigenetic tumor classification and shows that DNA methylation-based classification can be achieved with limited tumor cells, such as FNA cytology samples. This suggests that the previously reported 13% misclassification rate of pancreatic neuroendocrine neoplasms using FNA cytology may be reduced with methylation profiling.^12^ The ability to assist in preoperative clinical decision making makes this diagnostic tool highly appealing.

DNA methylation is now routinely used to aid diagnostics of central nervous and soft tissue tumors.^15,18^ EPIC array can be performed on histologic and cytologic specimens, is routinely available in most academic centers or accessible on a consultation basis, and results are obtained in 1–2 weeks. Ongoing optimizations of brain tumor classifiers aim for a diagnosis within 90 minutes,^33^ offering promising prospects for broader clinical use. Considering our model is a proof-of-concept classification model, further extension and validation are necessary before considering its widespread accessibility for daily practice.

To conclude, this study presents a highly accurate DNA methylation-based classifier capable of differentiating pancreatic neoplasms. At the same time, non-pancreatic entities are identified with near perfect precision, thereby preventing false pancreatic tumor diagnoses excellently. The successful classification of FNA samples makes the technique applicable in the preoperative setting.

## Supplementary Material

1

Note: To access the supplementary material accompanying this article, visit the online version of *Clinical Gastroenterology and Hepatology* at www.cghjournal.org, and at http://doi.org/10.1016/j.cgh.2024.02.007.

## Figures and Tables

**Table 1.** Overview of Pancreatic Dataset Including Source, Dataset Number, Tissue Type, and Array Type

Source	Dataset	Tissue type	Array	Tumor type							
				PDAC	PNET	PNEC	ACC	SPN	PB	NORM	Total
Benhamida et al^22^	NA	FFPE	EPIC	0	0	0	7	0	16	0	23
Jäkel et al^23^	EGAS00001002533	FFPE	450K	0	17	0	37	0	0	6	60
Chan et al^24^	GSE117852	FF	450K	0	23	0	0	0	0	0	23
Di Domenico et al^25^	EMTAB-7924	FFPE	450K	0	46	0	0	0	0	14	60
Endo et al^26^	GSE155353	FF	450K	82	0	0	0	0	0	0	82
Selenica et al^27^	NA	FFPE	EPIC	0	0	0	0	13	0	0	13
Yachida et al^28^	JGAS000359	Unknown	EPIC	0	30	13	0	0	0	0	43
Local cohort	EGAS00001004878	FFPE	EPIC	0	8	0	2	7	0	0	17
				82	124	13	46	20	16	20	321

ACC, acinar cell carcinoma; FF, fresh frozen; FFPE, formalin-fixed, paraffin-embedded; NA, not applicable; NORM, normal pancreatic tissue; PB, pancreatoblastoma; PDAC, pancreatic ductal adenocarcinoma; PNEC, pancreatic neuroendocrine carcinoma; PNET, pancreatic neuroendocrine tumor; SPN, solid pseudopapillary neoplasm.

## What You Need to Know

### Background

Non-ductal pancreatic neoplasms have overlapping cytological, histological and immunohistochemical features, which often makes correct histopathological diagnosis challenging, while it is crucial for prognosis and therapeutic decision making.

### Findings

Methylation profiling enables highly accurate classification of pancreatic neoplasms and near perfect identification of non-pancreatic entities, and can also be applied in fine-needle-aspiration / biopsy specimens, providing a valuable adjunct for pre-operative diagnostics.

### Implications for patient care

This DNA methylation-based classifier can reduce (pre-operative) misdiagnoses and thereby assist in pre-operative clinical decision making, enhancing therapy accuracy, and improving patient selection for studies. Prospective studies are needed to further validate and potentially extend the model.

## Abbreviations used in this paper

ACC: acinar cell carcinoma
FNA: fineneedle aspirate
MiNEN: mixed neuroendocrine–nonneuroendocrine neoplasm
NN: neural network
PB: pancreatoblastoma
PDAC: pancreatic ductal adenocarcinoma
PNEC: pancreatic neuroendocrine carcinoma
PNET: pancreatic neuroendocrine tumor
RF: random forest
SPN: solid pseudopapillary neoplasm
UMAP: uniform manifold approximation and projection
XGB: extreme gradient boosting
