# Supplementary material for: DNA Methylation Profiling Enables Accurate Classification of Nonductal Primary Pancreatic Neoplasms
Source: Clin Gastroenterol Hepatol. Author manuscript; Available in PMC 2026 Aug 18. (PMC13482779; doi:10.1016/j.cgh.2024.02.007)
Supplement: Supplementary file 1 [file NIHMS2200881-supplement-1.pdf]

Supplementary Methods

Patients and Samples

Methylation data for the pancreatic dataset (n = 321) were obtained from 7 previously published datasets (n = 304)[1–7] and from resection specimens collected at the University Medical Center Utrecht (UMCU) and Radboud University Medical Center (Radboud UMC), The Netherlands (n = 17) (Table 1). Dataset details can be found in Table 1. Datasets used in Selenica et al[6] and Behamida et al[1] were obtained from the authors on request. Data collected from the UMCU and Radboud UMC pathologic archives were obtained with approval by the UMCU and Radboud UMC Biobank Research Ethics Committees. The data that have not been deposited at EGA, which is hosted by the European Bioinformatics Institute and the Centre for Genomic Regulation under accession number EGAS00001004878,[8] are available on reasonable request. Methylation data of the pancreatic dataset were generated on Infinium HumanMethylation450 (n = 225) and Infinium HumanMethylationEPIC v1.0 (n = 96) platforms.

To facilitate detection of nonpancreatic entities, nonpancreatic tumor samples (n = 3708), spanning 29 different tumor types, were obtained from The Cancer Genome Atlas (TCGA). Three cases, referred for a second opinion or treatment at our tertiary center, which were histopathologically difficult and/or misdiagnosed, were used to illustrate the added value of the classifier (Figure 5). The cases comprise 2 primary tumors included in the pancreatic dataset and 2 additional metastatic cases, 1 of which was a matched metastasis from case 1 (Supplementary Table 1).

The additional analysis on (1) MiNENs (n = 7) included 1 additional local MiNEN case and 6 samples from Benhamida et al[1]; (2) matched primary and metastatic cases (n = 5) included 3 primary tumors included in the pancreatic dataset, 1 primary tumor used for MiNEN analysis, and 5 additional matched metastatic cases, all were local cases; and (3) matched FNA and resection specimens (n = 4) of which all 4 resection specimens were included in the pancreatic dataset, all were local cases (Supplementary Table 1).

Methylation Array Processing

DNA extraction and bisulfite conversion were conducted at our in-house core facility. Freshly cut unstained (4 μm) sections were used and tumor tissue was macrodissected for DNA extraction. DNA extraction was performed using the Maxwell RSC FFPE Plus DNA kit (Promega) after 2-hour proteinase K digestion. Subsequent steps adhered to protocols from the DNA Methylation BeadChip manufacturer (Illumina). In brief, EZ DNA Methylation Kit (Zymo Research) was used for bisulfite conversion, and FFPE Restore Kit (Illumina) addressed formalin fixation-induced DNA damage. Whole-genome amplification on the bisulfite converted and restored DNA was performed, followed by fragmentation, precipitation, and hybridization to CpG Site–specific Beads (Illumina). After clearing unhybridized and nonspecifically hybridized DNA, BeadChips were extended, stained, coated, and scanned using the iScan Software (Illumina). Raw data were exported as IDAT files for further processing.

Preprocessing

IDAT files were analyzed using the software package *minfi*[9] and normalized using the *preprocessNoob* function.[10] Next, probes were removed that (1) were located on sex chromosomes, (2) contain single-nucleotide polymorphisms in the CpG or single-base extension site, or (3) were found to exhibit cross-reactivity previously.[11] Finally, data were filtered for probes available on both platforms (n = 400,962), and available beta values were merged into a data table. IDAT files for TCGA samples were downloaded from https://portal.gdc.cancer.gov; processed as outlined previously; and filtered for primary, nonpancreatic neoplasms (n = 3708 remaining).

Quality Control and Batch Effect

Average methylation (beta value) was calculated for all filtered probes. Bisulfite conversion scores were calculated by dividing the minimal intensity for the red channel by the maximum intensity of the green channel for Bisulfite Conversion II control probes. Tumor purity was predicted using the Bioconductor package *RFpurify*.[12] Batch effect was evaluated by analyzing tissue types that were obtained from 2 or more studies. Average methylation values, conversion scores, and tumor purities were compared, and graphically inspected by *UMAP*.[13]

Unsupervised Analysis

To improve clustering and because of computational considerations, unsupervised analysis was performed on a reduced dataset filtered for the n = 5000 most variable CpG sites across the training set (as ranked by variance). Unsupervised analysis using all probes did not reveal major differences in clustering (Supplementary Figure 1). The R package *umap* was used for dimensionality reduction with *UMAP* with n_neighbours = 15 and min_dist = 0.2.

Classifier Development and Validation

Figure 1*A* shows an overview of the classifier development. Samples were randomly assigned to the training and test cohort stratified by tumor type (50/50 ratio; Figure 3*A*) using the R package *caret*.[14] All models were trained on a reduced dataset using the 5000 most

variable probes as input with 10-fold cross-validation and n = 3 repetitions per fold. To account for uneven distribution across the different tumor types, the *upSample* function was used to upsample all classes to the same number of cases. Neural network, random forest and XGBoost algorithms were trained on the training cohort using the R package *Keras*,[15] *randomForest*,[16] and *xgboost*,[17] respectively. Samples of the test cohort were at no point used for variable selection or model building. Random forest model was trained with default parameters: *trees* = 500, *mtry* = sqrt(5000). XGBoost machines were trained with the following parameters: *nrounds* = 100, *max_depth* = 6, *eta* = 0.3, *gamma* = 1, *colsample_bytree* = 1, *min_child_weight* = 1, *subsample* = 1. The neural network model was trained for n = 50 epochs with a 4-layer structure. Layers 1–3 used 64 neurons with *relu* activation, the last layer *softmax* activation. *RMSProp* was used as the optimizer, *categorical crossentropy* as the loss function, and *accuracy* as the optimization metric. The highest score was used to assign samples to a class (*majority vote*). One-way analysis of variance was used to assess the difference in model accuracies, considering a $P < .05$ statistically significant. Classifier performance for different probability score thresholds was assessed by calculating the proportion of predictable cases and accuracy for every cutoff. Confusion matrices were calculated for all 3 models, and the clustering of misclassification of the best performing classifier was visualized using UMAP.

Figure 1*B* shows an overview of the detection of nonpancreatic entities. First, the best performing model's classification results were assessed in an extended cohort of n = 3708 nonpancreatic neoplasms. Second, all samples of the TCGA and pancreatic cohorts were randomly assigned to a training and test set (50/50 split). A logistic regression model using the score and label of the best performing model was trained to differentiate between pancreatic and nonpancreatic (ie, outlier) samples and evaluated in the test set. Finally, model performance in terms of accuracy and drop-out was calculated for different thresholds of logistic regression probability scores. Student *t* test was used to compare random forest model probability scores distribution between the pancreatic and nonpancreatic cohorts. A $P < .05$ was considered statistically significant.

Additional Analysis

Furthermore, we performed 3 additional subanalyses with (1) MiNEN (n = 7), (2) matched primary and metastatic cases (n = 5), and (3) matched FNA and resection specimens (n = 4). The MiNEN tumors' relationship to other tumor types using reduced dataset (n = 5000 most variable CpG sites) was visualized using UMAP, and entropy scores and mean probability scores per tumor type were evaluated. For the matched primary and metastatic cases and matched FNA and resection specimens, correlations between classifier probability scores in comparison with nonmatched cases were evaluated.

References

1. Benhamida JK, Vyas M, Tanaka A, et al. Pancreatoblastomas and mixed and pure acinar cell carcinomas share epigenetic signatures distinct from other neoplasms of the pancreas. Mod Pathol 2022;35:956–961.
2. Chan CS, Laddha SV, Lewis PW, et al. ATRX, DAXX or MEN1 mutant pancreatic neuroendocrine tumors are a distinct alpha-cell signature subgroup. Nat Commun 2018;9:4158.
3. Di Domenico A, Pipinikas CP, Maire RS, et al. Epigenetic landscape of pancreatic neuroendocrine tumours reveals distinct cells of origin and means of tumour progression. Commun Biol 2020;3:740.
4. Endo Y, Fujimoto M, Ito N, et al. Clinicopathological impacts of DNA methylation alterations on pancreatic ductal adenocarcinoma: prediction of early recurrence based on genome-wide DNA methylation profiling. J Cancer Res Clin Oncol 2021;147:1341–1354.
5. Jakel C, Bergmann F, Toth R, et al. Genome-wide genetic and epigenetic analyses of pancreatic acinar cell carcinomas reveal aberrations in genome stability. Nat Commun 2017;8:1323.
6. Selenica P, Raj N, Kumar R, et al. Solid pseudopapillary neoplasms of the pancreas are dependent on the Wnt pathway. Mol Oncol 2019;13:1684–1692.
7. Yachida S, Totoki Y, Noe M, et al. Comprehensive genomic profiling of neuroendocrine carcinomas of the gastrointestinal system. Cancer Discov 2022;12:692–711.
8. Hackeng WM, Dreijerink KMA, de Leng WWJ, et al. Genome methylation accurately predicts neuroendocrine tumor origin: an online tool. Clin Cancer Res 2021;27:1341–1350.
9. Aryee MJ, Jaffe AE, Corrada-Bravo H, et al. Minfi: a flexible and comprehensive bioconductor package for the analysis of Infinium DNA methylation microarrays. Bioinformatics 2014;30:1363–1369.
10. Fortin JP, Triche TJ Jr, Hansen KD. Preprocessing, normalization and integration of the Illumina HumanMethylationEPIC array with minfi. Bioinformatics 2017;33:558–560.
11. Chen YA, Lemire M, Choufani S, et al. Discovery of cross-reactive probes and polymorphic CpGs in the Illumina Infinium HumanMethylation450 microarray. Epigenetics 2013;8:203–209.
12. Johann PD, Jager N, Pfister SM, et al. RF_Purify: a novel tool for comprehensive analysis of tumor-purity in methylation array data based on random forest regression. BMC Bioinformatics 2019;20:428.
13. McInnes L, Healy J, Melville J. UMAP: Uniform Manifold Approximation and Projection for Dimension Reduction. ArXiv e-prints 2018 1802;03426.
14. Kuhn M. Building predictive models in R using the caret package. J Stat Softw 2008;28:1–26.
15. J. Allaire FC. R interface to keras. Available at: https://github.com/rstudio/keras. Accessed March 26, 2024.
16. Breiman L. Random forests. Mach Learn 2001;45:5–32.
17. Chen CQ, Guestrin C. Xgboost: A Scalable Tree Boosting System:Proceedings of the 22nd ACM SIGKDD International Conference on Knowledge Discovery and Data Mining, San Francisco, 13-17 August 2016, 785-794; 2016.

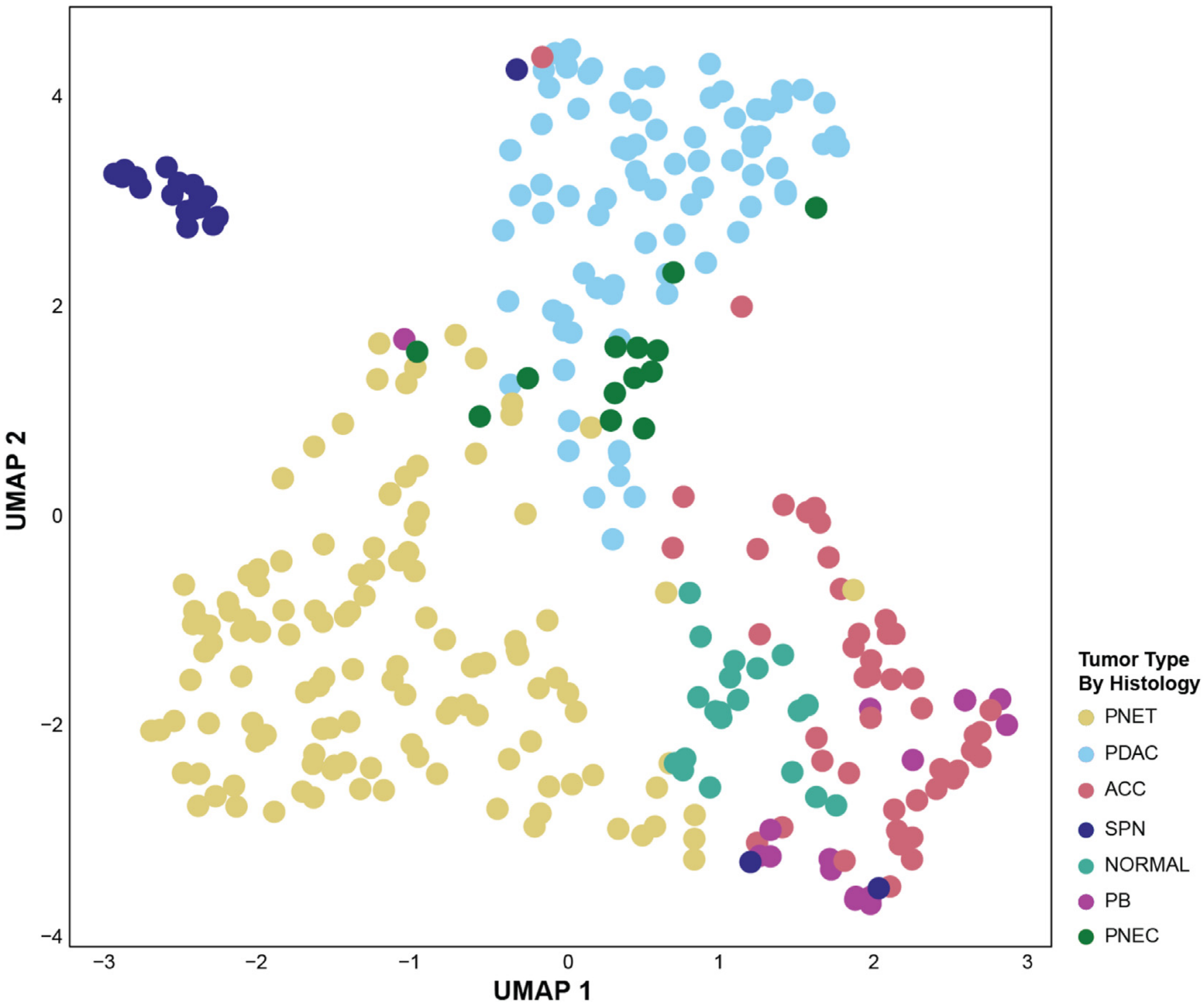

Supplementary Figure 1. Primary pancreatic dataset projected onto the UMAP using all probes.

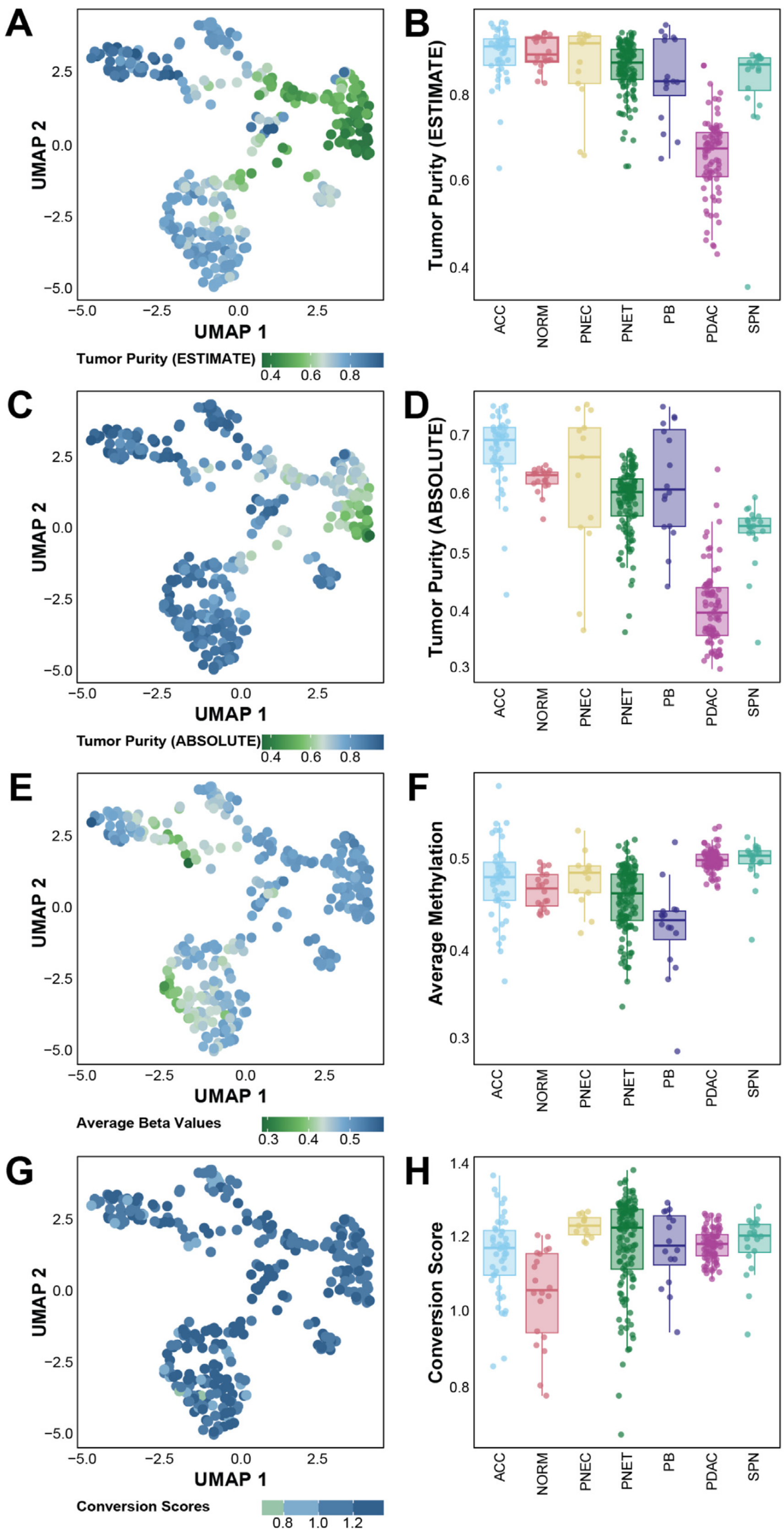

Supplementary Figure 2. Additional unsupervised analysis of pancreatic dataset. ESTIMATE and ABSOLUTE tumor cell purity using the DNA methylation-based "RFPurify" method projected over UMAP and per pancreatic tumor type showing lower tumor purity for pancreatic ductal adenocarcinoma compared with other tumor types (*A-D*). Average methylation and conversion score projected over UMAP and per pancreatic tumor type showing lower genome-wide methylation in pancreatoblastoma (*E-H*).

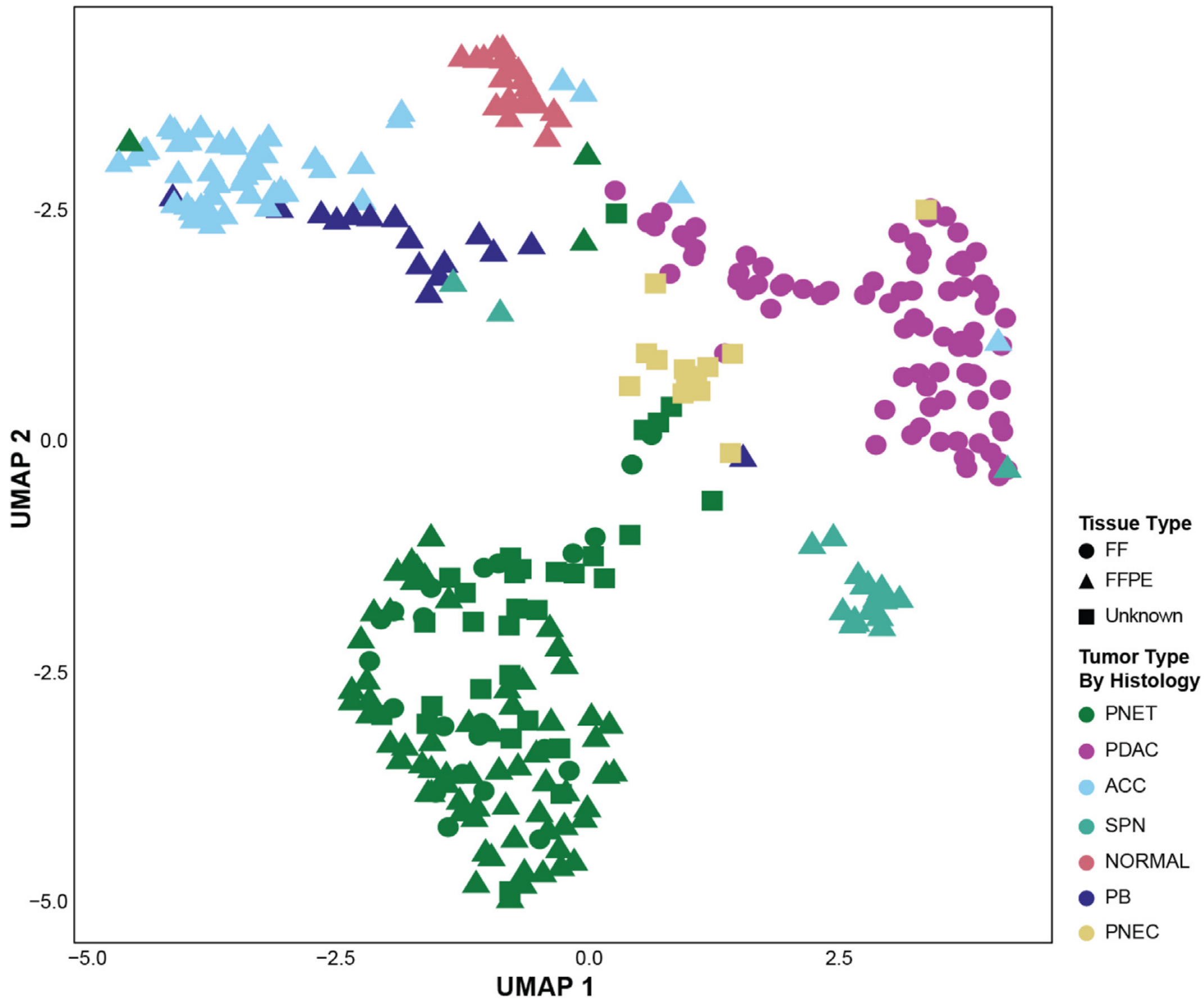

Supplementary Figure 3. Tissue types were projected onto the UMAP. Showing no difference in clustering between fresh-frozen (FF) and formalin-fixed, paraffin-embedded (FFPE) tissue.

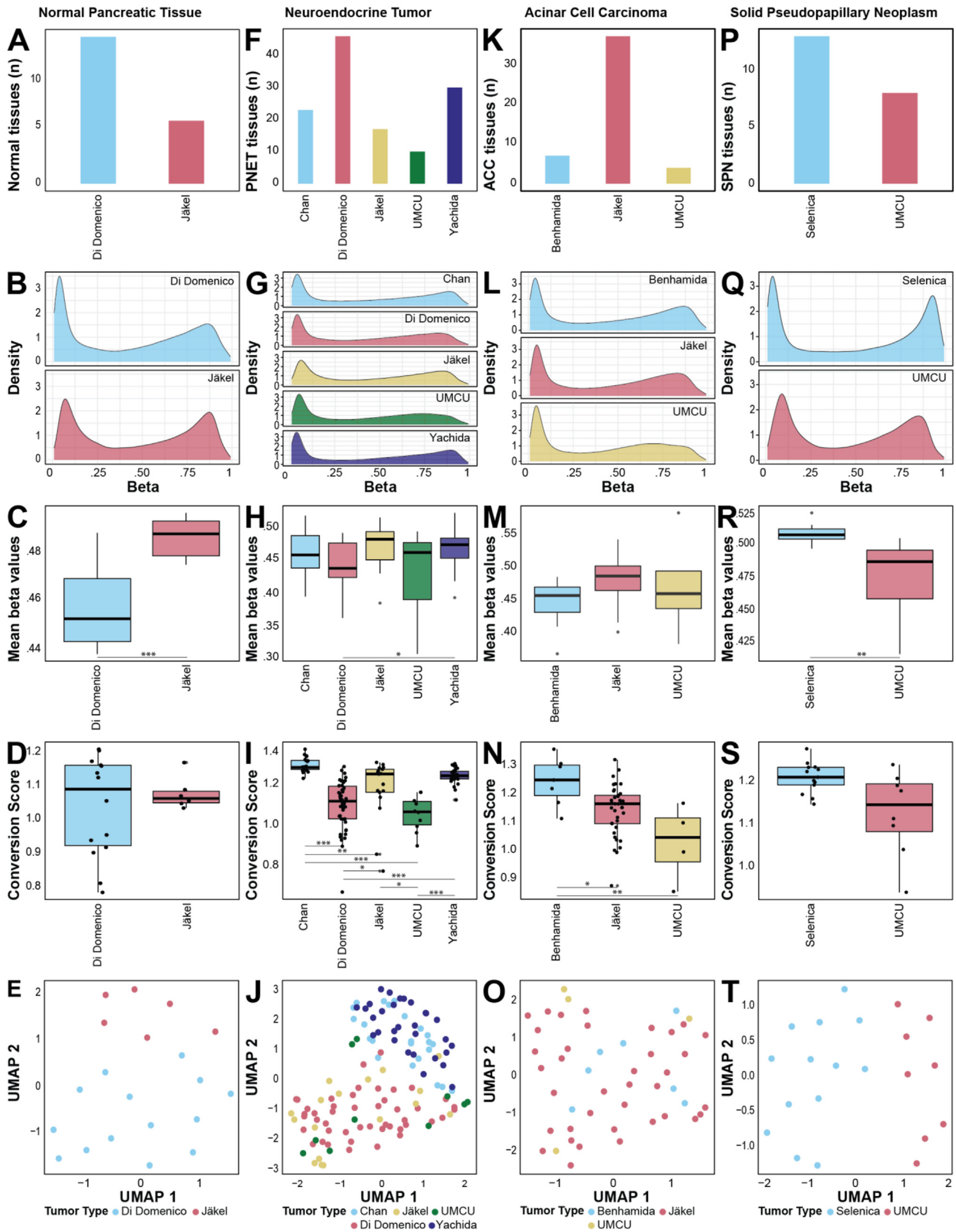

Supplementary Figure 4. Batch effect analysis. Number of samples per source, density plots, mean beta values, conversion scores, and dimensionality reduction through *UMAP* for normal pancreatic tissue (*A-E*), pancreatic neuroendocrine tumor (*F-J*), acinar cell carcinoma (*K-O*), and solid pseudopapillary neoplasm (*P-T*). Results show some differences between sources in the density plots, mean beta values, conversion scores, and UMAP across tumor types. Batch effect analyses revealed small but consistent differences regarding beta values distribution and conversion scores across tumor types. This effect was strongest in normal pancreatic and solid pseudopapillary neoplasm samples, and may suggest batch effects. However, classifier performance seems not to be influenced by the origin of a sample. This implies robustness of the classifier toward minor technical differences resulting from specimen selection and reagent handling. *$P < .05$; **$P < .01$; ***$P < .001$.

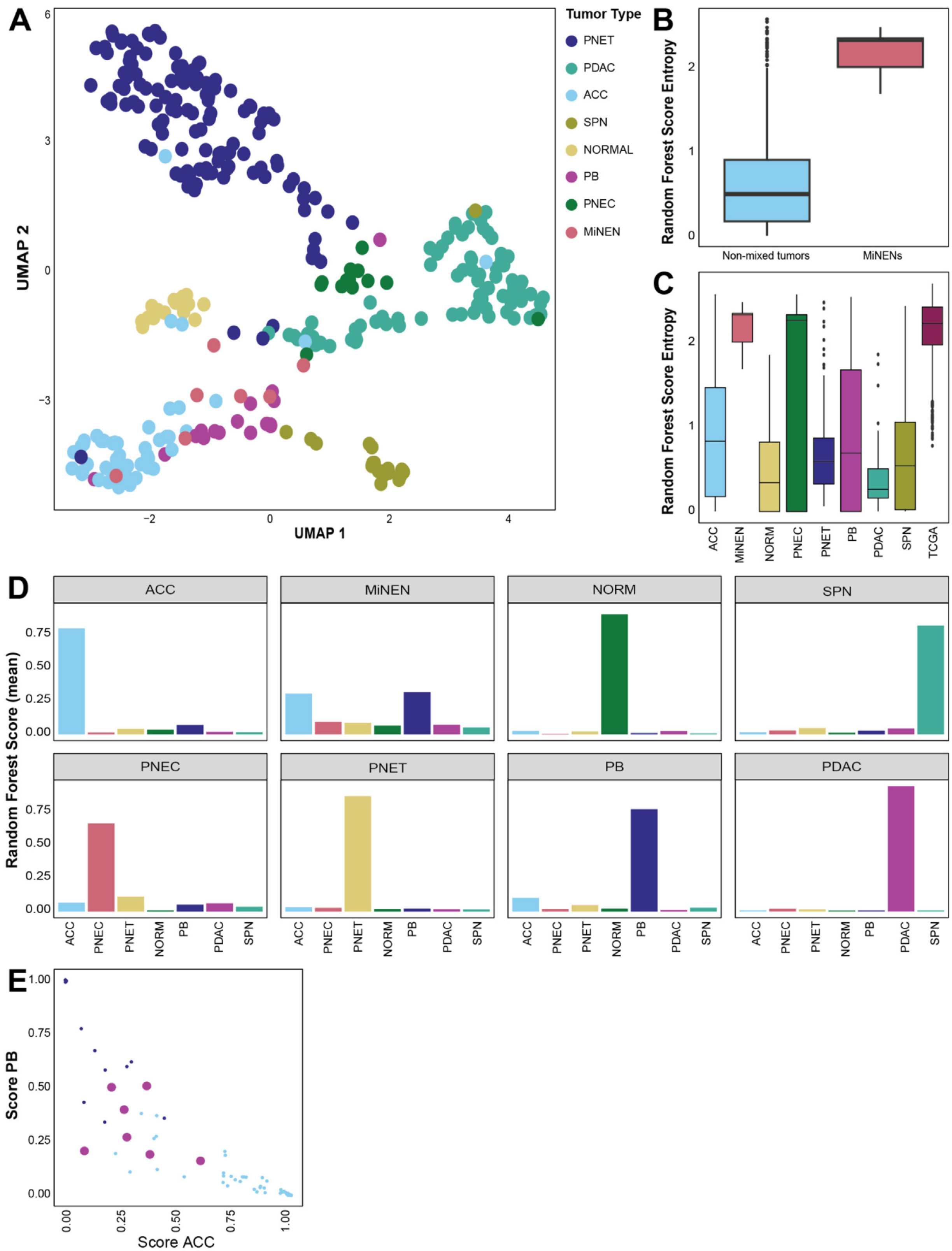

Supplementary Figure 5. Analysis of MiNEN. Dimensionality reduction through *UMAP* showing (n = 7) MiNENs samples tend to cluster with acinar cell carcinomas and pancreatoblastomas (*A*). Random **forest** model probability scores of MiNENs show higher entropy as compared with nonmixed tumors (*B*). Random forest model probability scores of MiNENs and TCGA samples show higher entropy as compared with primary pancreatic neoplasms (*C*). Distribution of the mean random forest model probability scores show high scores for the corresponding tumor type, and divergent scores for MiNEN (*D*). Comparing the MiNENs probability scores for acinar cell carcinoma and pancreatoblastoma with that of acinar cell carcinoma and pancreatoblastoma, shows that MiNENs fall between pancreatoblastoma and acinar cell carcinoma (*E*).

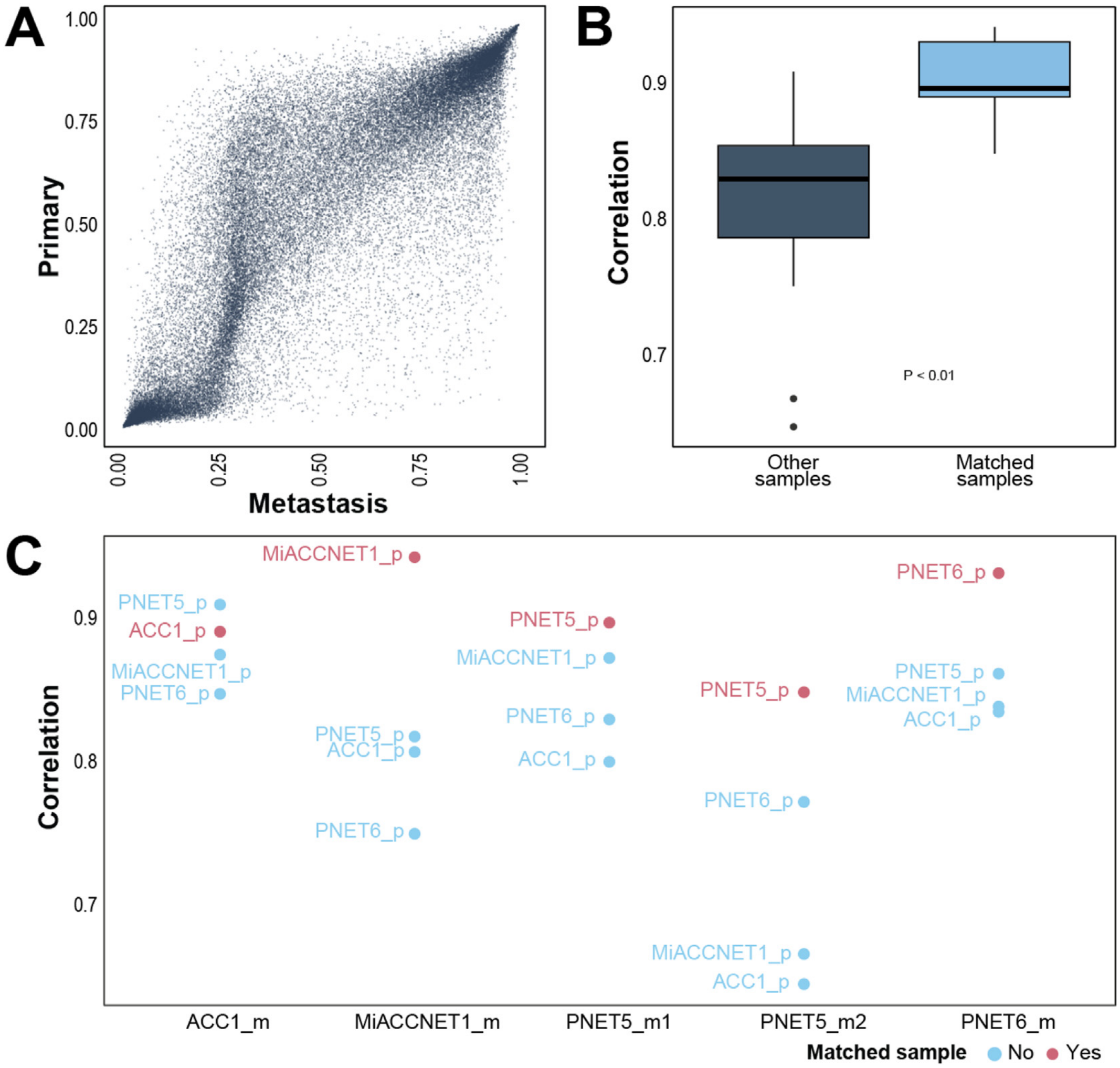

Supplementary Figure 6. Analysis of matched primary and metastatic cases. Comparison of the unfiltered beta values of the primary and matched metastasis cases show some correlation (*A*). When quantifying the correlation between the beta values a significantly higher correlation between beta values of matched cases as compared with the nonmatched cases is seen (paired T-test: $P < .01$; 95% confidence interval, -0.15 to -0.04) (*B*). When analyzing the correlation between the samples, it becomes apparent that in most cases the matched primary sample shows superior correlation to its matched metastases sample as compared with other cases. The metastatic acinar cell carcinoma (Figure 5, case 1) shows highest correlation with a primary PNET sample (*C*).

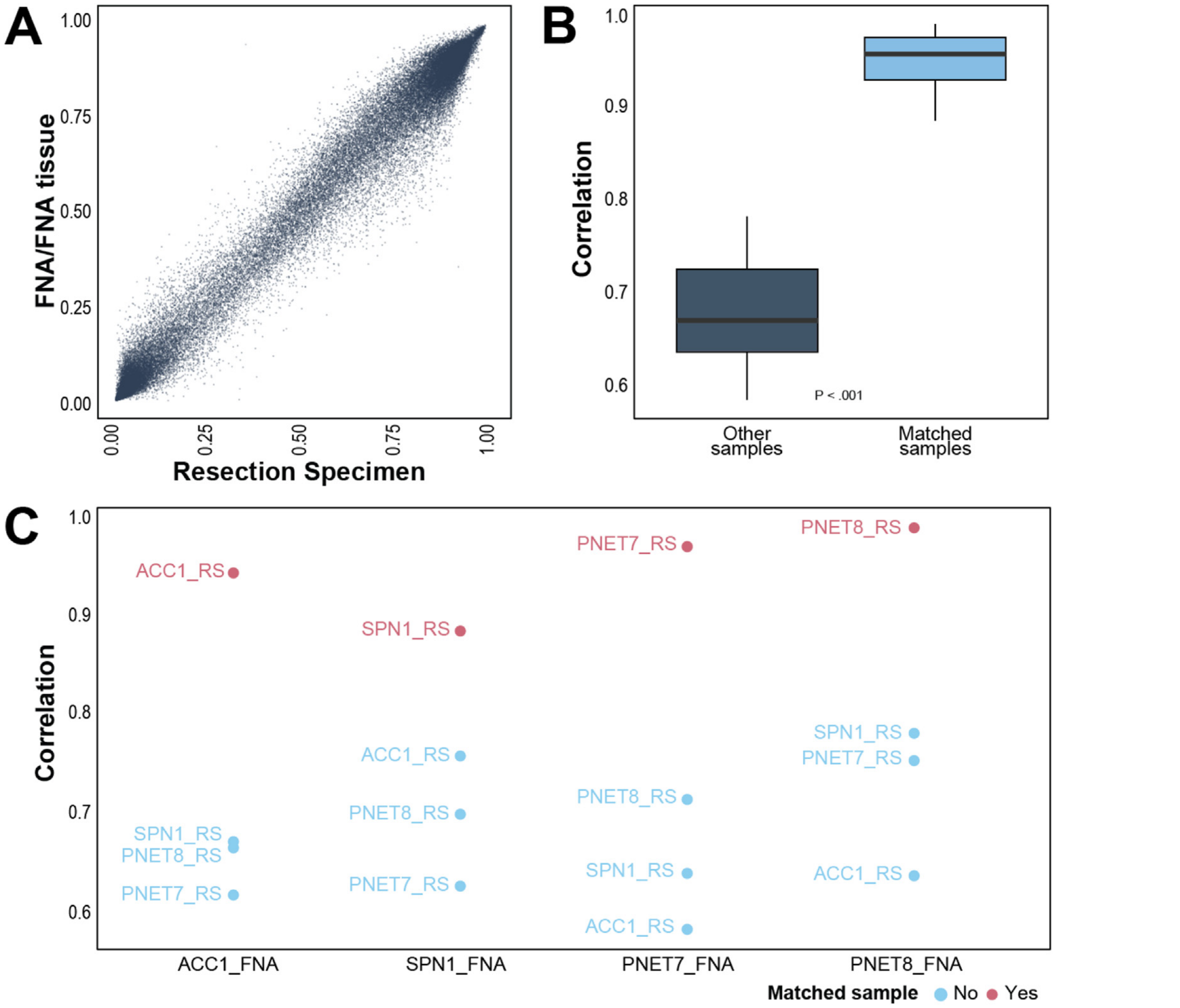

Supplementary Figure 7. Analysis of matched FNA and resection specimen cases. The unfiltered beta values of the FNA and resection specimen cases show high correlation (*A*). When quantifying the correlation between the beta values a significant higher correlation between beta values of matched cases as compared with the nonmatched cases is seen (paired T-test $P <$ .001; 95% confidence interval, -0.33 to -0.20) (*B*). When analyzing the correlation between the samples, it becomes apparent that in all instances the resection specimen samples show superior correlation to their matched FNA sample as compared with other cases (*C*).

Supplementary Table 1. Overview of Histopathologic Challenging and Misdiagnosed Clinical Cases and Samples Used Additional Analysis Including, Source, Array Type, Tumor Type, Location, and Sample Type

Source	Sample name	Array type	Tumor type	Location	Sample Type	In pancreatic dataset
Histopathologic challenging and misdiagnosed clinical cases (Figure 5)						
UMCU	UMCU_ACC1_M	EPIC	ACC	Bone metastasis	FNB	No
UMCU	UMCU_ACC1	EPIC	ACC	Primary	Resection	Yes
UMCU	UMCU_SPN1	EPIC	SPN	Primary	Resection	Yes
UMCU	UMCU_ACC2_M	EPIC	ACC	Liver metastasis	Resection	No
Analysis on MiNEN						
UMCU	UMCU_MiACCNET1	EPIC	MNEN	Primary	Resection	No
Benhamida	Mixed_ACC1	EPIC	MiNEN	Primary	Resection	No
Benhamida	Mixed_ACC2	EPIC	MiNEN	Primary	Resection	No
Benhamida	Mixed_ACC3	EPIC	MiNEN	Primary	Resection	No
Benhamida	Mixed_ACC4	EPIC	MiNEN	Primary	Resection	No
Benhamida	Mixed_ACC5	EPIC	MiNEN	Primary	Resection	No
Benhamida	Mixed_ACC6	EPIC	MiNEN	Primary	Resection	No
Analysis on matched primary and metastatic cases						
UMCU	UMCU_panNET6	EPIC	PNET	Primary	Resection	Yes
UMCU	UMCU_panNET6_M	EPIC	PNET	Liver metastasis	Resection	No
UMCU	UMCU_panNET5	EPIC	PNET	Primary	Resection	Yes
UMCU	UMCU_panNET5_M1	EPIC	PNET	Liver metastasis	FNB	No
UMCU	UMCU_panNET5_M2	EPIC	PNET	Liver metastasis	Resection	No
UMCU	UMCU_ACC1	EPIC	ACC	Primary	Resection	Yes
UMCU	UMCU_ACC1_M	EPIC	ACC	Bone metastasis	FNB	No
UMCU	UMCU_MiACCNET1	EPIC	MiNEN	Primary	Resection	No
UMCU	UMCU_MiACCNET1_M	EPIC	MiNEN	Metastasis	FNA	No
Analysis on matched FNA and resection specimens						
UMCU	UMCU_panNET7_b	EPIC	PNET	Primary	FNA	No
UMCU	UMCU_panNET7	EPIC	PNET	Primary	Resection	Yes
UMCU	UMCU_panNET8_b	EPIC	PNET	Primary	FNA	No
UMCU	UMCU_panNET8	EPIC	PNET	Primary	Resection	Yes
RB	RB_ACC1_b	EPIC	ACC	Primary	FNA	No
RB	RB_ACC1	EPIC	ACC	Primary	Resection	Yes
RB	RB_SPN1_b	EPIC	SPN	Primary	FNA	No
RB	RB_SPN1	EPIC	SPN	Primary	Resection	Yes

ACC, acinar cell carcinoma; FNA, fine-needle aspiration; FNB, fine-needle biopsy; MiNEN, mixed neuroendocrine–nonneuroendocrine neoplasm; PNET, pancreatic neuroendocrine tumor; RB, Radboud UMC; SPN, solid pseudopapillary neoplasm; UMCU, University Medical Center Utrecht.
